# Postoperative Intensive Care Management of Aortic Repair

**DOI:** 10.3390/jpm12081351

**Published:** 2022-08-22

**Authors:** Stefano De Paulis, Gabriella Arlotta, Maria Calabrese, Filippo Corsi, Temistocle Taccheri, Maria Enrica Antoniucci, Lorenzo Martinelli, Francesca Bevilacqua, Giovanni Tinelli, Franco Cavaliere

**Affiliations:** 1 Fondazione Policlinico Gemelli IRCCS, 00168 Rome, Italy; 2 Department of Cardiovascular Sciences, Università Cattolica del Sacro Cuore, 00168 Rome, Italy

**Keywords:** aortic repair, postoperative complications, intensive care management

## Abstract

Vascular surgery patients have multiple comorbidities and are at high risk for perioperative complications. Aortic repair surgery has greatly evolved in recent years, with an increasing predominance of endovascular techniques (EVAR). The incidence of cardiac complications is significantly reduced with endovascular repair, but high-risk patients require postoperative ST-segment monitoring. Open aortic repair may portend a prohibitive risk of respiratory complications that could be a contraindication for surgery. This risk is greatly reduced in the case of an endovascular approach, and general anesthesia should be avoided whenever possible in the case of endovascular repair. Preoperative renal function and postoperative kidney injury are powerful determinants of short- and long-term outcome, so that preoperative risk stratification and secondary prevention are critical tasks. Intraoperative renal protection with selective renal and distal aortic perfusion is essential during open repair. EVAR has lower rates of postoperative renal failure compared to open repair, with approximately half the risk for acute kidney injury (AKI) and one-third of the risk of hemodialysis requirement. Spinal cord ischemia used to be the most distinctive and feared complication of aortic repair. The risk has significantly decreased since the beginning of aortic surgery, with advances in surgical technique and spinal protection protocols, and is lower with endovascular repair. Endovascular repair avoids extensive aortic dissection and aortic cross-clamping and is generally associated with reduced blood loss and less coagulopathy. The intensive care physician must be aware that aortic repair surgery has an impact on every organ system, and the importance of early recognition of organ failure cannot be overemphasized.

## 1. Introduction

Vascular surgery patients have multiple comorbidities and are at high risk for perioperative complications. Surgery of the aorta is characterized by major hemodynamic changes and significant blood loss that contribute to overall morbidity. Perioperative management requires accurate preoperative assessment, optimal intraoperative anesthetic management and attentive postoperative care. Postoperative intensive care unit (ICU) management should aim at organ function support and early detection of complications. Aortic repair surgery has greatly evolved in recent years with an increasing predominance of endovascular techniques. This evolving context is reshaping the pattern and incidence of postoperative complications and the ICU management strategy.

## 2. Cardiovascular Complications

### 2.1. Open Repair

Open aortic repair surgery (descending thoracic, thoracoabdominal and abdominal aorta) is burdened with several cardiovascular complications, the most serious being myocardial ischemia and infarction [1,2]. Perioperative myocardial infarction (MI) is defined as an increase and/or decrease in cardiac troponin (cTn) with at least one value above the 99th percentile upper reference limit, with at least one of the symptoms of myocardial ischemia (new ischemic electrocardiographic (ECG) changes, new pathological Q waves on ECG or imaging evidence of new regional wall motion abnormalities) [3]. 

In total, 3.6% of patients undergoing major vascular procedures sustain a diagnosis of perioperative MI, and 2.9–23.9% of the patients, depending on the cardiac biomarker measured, have evidence of myocardial injury in the first three days after surgery [4]. Analyzing the timing of biomarkers’ elevation in 1136 patients after abdominal aortic surgery, two different pathophysiologic mechanisms have been postulated: in 2% of patients, cTn elevation in the first 24 h after surgery was probably caused by an acute thrombotic coronary occlusion consequent to a vulnerable plaque rupture; in 3% of patients, MI occurred 24–72 h after surgery preceded by a prolonged period of increased cTn values and was probably related to an imbalance between myocardial oxygen supply and demand caused by several factors that are present after major surgery, such as thrombogenicity, high levels of circulating tissue factor and sympathetic overactivity [5]. These patients also had a significantly increased short- and long-term mortality: much higher in patients with MI compared to patients with isolated biomarker elevation [4]. A meta-analysis confirms that postoperative cTn elevation occurred in 15.5% of patients following vascular surgery [6]. The 30-days mortality was different between three groups: 2.3% for patients with no rise of troponin, 11.6% for patients with an isolated increase of troponin and 21.6% for patients with a documented MI. Hypothermic patients (core temperature <35 °C), after major vascular surgery, have a greater risk of myocardial ischemia in comparison to normothermic patients [7]. 

New-onset postoperative arrhythmias are another cardiovascular complication after vascular surgery. The reported incidence of postoperative arrhythmias in the general surgical population ranges from 0.37% for atrial fibrillation after noncardiac, nonthoracic surgery to 20%, varying among different studies because of the population characteristics, type of surgery and ECG detection method [8]. In 513 vascular surgical patients, 11% developed new-onset ventricular (4%) and supraventricular (7%) tachyarrhythmias in the perioperative period; in the postoperative period, arrhythmias occurred in 5.6% of patients, 8.3 (0.9–10.2) hours after the end of surgery and 97% of patients were asymptomatic [9]. In the group of patients that underwent open abdominal aortic aneurysm (AAA) repair, the incidence of perioperative arrhythmias was 14.5%. The incidence was higher among elderly patients and those with reduced left ventricular ejection fraction (LVEF) [9]. In this context, myocardial stretch can induce myocyte depolarization, causing abnormal impulses that trigger arrhythmias. As for other noncardiac surgical patients, the pathophysiological mechanism of arrhythmias may be related to electrolyte derangement, but more frequently to the activation of inflammatory pathways [10] and to the increased sympathetic and hormonal activity which characterizes the postoperative period [11,12]. Arrhythmias are an independent predictor of cardiovascular events and death [9]. Supraventricular and ventricular tachyarrhythmias may increase myocardial oxygen demand and reduce ventricular filling time resulting in hypotension and hypoperfusion. 

Other common, adverse postoperative cardiovascular events after vascular surgery are acute hypertension or hypotension. Acute hypertensive crises are related to increased sympathetic tone and vascular resistance and may be related to pain, cold, hypoxemia and hypercarbia [13]. Hypertension usually begins 10–20 min after surgery and lasts up to 4 h and, if untreated, it may cause bleeding by compromising suture lines and it may precipitate cerebrovascular accidents [14]. Moreover, hypertension increases ventricular wall tension and myocardial work, elevating myocardial oxygen demand and the risk of MI [15]. From the other side, hypotension is often due to hypovolemia or vasoplegia and it is associated with an increased incidence of multi organ failure, renal failure, graft thrombosis and gut or limb ischemia. 

Vascular complications include bleeding from aortic graft anastomoses and lower extremity ischemia. Ischemia occurs in 2–5% after open AAA repair because of distal atheromatous emboli [15]. 

### 2.2. Endovascular Repair

With endovascular repair, the incidence of cardiac complications is significantly reduced (14.6% vs. 32.1%; OR 0.37 (0.20–0.66), *p* 0.001) [16]. Among early vascular complications, vascular access injuries are the most frequent (15–20%) together with lower limb ischemia [15,17]. The use of large delivery catheters, inserted in a retrograde manner via the iliofemoral vessels, can cause arterial dissection, arterial perforation or iliac artery rupture; retrograde arterial dissection can cause mesenteric or renal ischemia and iliac artery laceration or rupture results in retroperitoneal hematoma or hemorrhage [17]. Stent graft migration, endoleaks with aneurysm expansion and rupture, stent graft infection and erosion into the esophagus are the most frequent late complications. 

### 2.3. Monitoring and Treatment 

To reduce the incidence of complications and their effect, early recognition and treatment is essential. Electrocardiographic monitoring using five or six leads placed in V3–V5 has an elevated predictive value; postoperative ST-segment monitoring is recommended for all patients undergoing open aortic repair and for high-cardiac risk patients having endovascular repair [18]. Postoperative troponin measurements are recommended for an early identification of myocardial ischemia for all patients with ECG changes or chest pain after surgery [18]. Rate and pressure control to reduce myocardial oxygen demand, associated with euvolemic balance and oxygen delivery optimization can reduce the incidence of myocardial ischemia. Complete and continuous hemodynamic monitoring of arterial and central venous pressure is recommended after open vascular surgery, whereas monitoring pulmonary arterial pressure by pulmonary artery catheter (PAC) should be reserved for cases at high risk of hemodynamic instability [18].

Transthoracic/transesophageal echocardiography to assess volume status and cardiac function is recommended in patients with reduced LVEF. The use of echocardiography to analyze cardiac wall motion abnormalities is a sensitive monitor of ischemia and is recommended in patients at high risk for MI [18].

The use of beta-blockers in high-risk patients and their administration in combination with statins decrease perioperative mortality [19,20]. Monitoring core temperature and the use of warmed inhaled gases and intravenous fluids to maintain body temperature above 36 °C appear to be beneficial in terms of hemodynamic stability and metabolic acidosis [18].

Tachyarrhythmias may cause hemodynamic instability and must be treated with antiarrhythmic drugs or electric cardioversion according to the current guidelines [21,22]. Diuresis and serum creatinine monitoring, together with lactate levels and hemoglobin and the distal pulse check, are essential for early recognition of vascular complications.

## 3. Respiratory Complications

Postoperative pulmonary complications (PPC) are severe conditions strongly associated with increased hospital morbidity, mortality and costs. PPC include pneumonia; respiratory tract infection; respiratory failure defined as mechanical ventilation for longer than 48 h; or unplanned reintubation, atelectasis, acute respiratory distress syndrome (ARDS), pulmonary edema, pleural effusion, hypoxia and other unspecified respiratory disorders.

Risk factors for PPC are age, poor functional status, history of cigarette smoking, obstructive pulmonary disease (COPD) or other intrinsic lung disease, congestive heart failure (CHF), diabetes and renal failure. Most patients undergoing vascular surgery belong to these categories and are at higher risk of developing pulmonary complications [23]. It is part of the preoperative evaluation to identify those patients and optimize multiple aspects of their care, including functional status (with lifestyle changes like improved nutrition and weight loss) and pulmonary function (either through enhanced medical therapy with bronchodilators or with smoking cessation or physiotherapy) [15].

Intraoperative risk factors include surgical division of the diaphragm, prolonged one-lung ventilation and massive blood products transfusion. The surgical site is one of the most important risk factors associated with the development of PPC. Abdominal aortic surgery, together with upper abdominal and thoracic procedures carry the highest risk [24,25,26].

In aortic repair surgery, postoperative pulmonary dysfunction may be a consequence of ischemia-reperfusion injury that is characterized by nonspecific alveolar damage, lung edema and hypoxemia occurring within 72 h after surgery [27]. In experimental studies, inflammatory cytokines were found to mediate neutrophil chemoattraction within the lung with endothelial cell swelling, capillary leak and edema, and after aortic surgery, the increased plasma concentration of IL-6 was correlated with increased protein permeability in the lungs [28]. The oxidative stress in the lungs, resulting from ischemia, causes the release of reactive oxygen species (ROS) mediators and lipid peroxidation. A burst of ROS appears immediately after reperfusion. They can react with nitric oxide to produce reactive nitrogen species, such as peroxynitrite, which are highly destructive radicals. Cellular injury leads to a rapid remodeling of the membrane lipids and the generation of bioactive lipid mediators. Phospholipases, in particular, phospholipase A2 (PLA2), induce the activation of platelet-activating factor and the degradation of arachidonic acid. This produces tromboxanes, prostaglandins and leukotrienes that stimulate leucocyte activation and chemotaxis [29].

Given the high-risk nature of patients with vascular disease, there is a significant difference between open and endovascular procedures. In fact, the pulmonary morbidity rate reported in one study was 3% in the endovascular group, compared with 15% in the open repair group, with a relative reduction of more than 80% in favor of endovascular procedures [23]. According to Brovman et al. [30], even though the average unplanned reintubation rate after all vascular surgery procedures was 2.2%, there was a significant variability in reintubation rates among different types of surgeries. In this study, a multivariate analysis showed an increased likelihood of reintubation for several procedures: abdominal aortic bypass (OR 4.5), axillary-femoral bypass (OR 2.1), excision abdominal graft (OR 5.4), open abdominal aortic aneurysm repair (OR 3.4), open thoracic aorta (OR 5.4), thoracic endovascular aneurysm repair (OR 3.4) and endovascular aneurysm repair (OR 0.9). Reintubated patients had significantly longer surgery and anesthesia times [31]. Patients undergoing thoracic, abdominal and open procedures were up to five times more likely to have an unplanned postoperative reintubation [30]. Based on these results, intraoperative anesthetic management should adopt processes of care and all the possible strategies to reduce the incidence of postoperative pulmonary complications. The use of lung protective ventilation, preservation of phrenic nerve function and diaphragmatic integrity may provide some postoperative pulmonary benefit.

EVAR was developed as a less invasive and potentially safer alternative to traditional open surgical repair for aortic aneurysm. Various anesthetic techniques can be applied to successfully accomplish EVAR, avoiding general anesthesia: epidural or spinal anesthesia and local infiltration anesthesia [32]. Surgical teams must discern in which patients this advantage can be most effectively used. Edwards et al. demonstrated significant advantages in the use of spinal and local anesthesia compared with general anesthesia in the performance of elective EVAR [32]. General anesthesia should be avoided whenever possible for EVAR [33]. Potential mechanisms by which locoregional anesthesia may affect the morbidity of EVAR include avoidance of endotracheal intubation and mechanical ventilation as well as the potential for residual neuromuscular paralysis after reversal. Locoregional anesthesia avoids mechanical ventilation maintaining spontaneous ventilation, thereby minimizing the patient’s exposure to factors that increase the risk of postoperative pulmonary failure. Locoregional techniques also allow the avoidance of ventilator weaning at the end of anesthesia, which can be challenging in compromised patients. Furthermore, locoregional anesthetic techniques provide preemptive analgesia and improve postoperative pain control compared to general anesthesia alone.

Postoperative strategies include head-up positioning, as part of the ventilator-associated pneumonia (VAP) prevention bundle and to reduce facial edema. After a long intraoperative period of one lung ventilation, prophylactic bronchoscopy improves left lung function before extubation. Prolonged mechanical ventilation is associated with increased pulmonary complications and perioperative mortality. Early extubation allows patient mobilization and minimizes the risk of infection with incentive spirometry. The application of nasal continuous positive airway pressure, with 10 cm H_2_O immediately after extubation, has been associated with fewer pulmonary complications by maintaining functional residual capacity and thus preventing atelectasis and hypoxemia, and reducing the work of breathing [34]. Along with the implementation of minimally invasive techniques and endovascular procedures, networking and cooperation between the surgeon, anesthesiologist, physiotherapist and the nursing team can lead to an optimization of perioperative care. Therefore, a “fast track” approach should be considered the gold standard in order to prevent the development of postoperative pulmonary complications.

## 4. Renal Complications

Patients undergoing vascular procedures are at a high risk of postoperative acute kidney injury (AKI) [35]. AKI is defined, according to the serum creatinine (SCr) based criteria of Kidney Disease Improving Global Outcomes (KDIGO), as a SCr increase of ≥26.5 μmol/L within 48 h, or a 1.5-fold increase in SCr within 7 days above the baseline value measured on admission [36,37].

Although various studies have used different criteria to define renal dysfunction, the incidence of postoperative AKI requiring dialysis ranges between 5% and 15%, and its occurrence is associated with a worse outcome [38,39], where preoperative renal dysfunction and severity of atherosclerotic disease are the most important preoperative predictors. In a recent systematic review [40], a univariate analysis showed several other preoperative predictors of renal failure including age over 75 years (OR = 1.58, 95% CI 1.11–2.26), treated hypertension (OR = 1.87, 95% CI 1.28–2.74), hyperlipidemia, preoperative serum creatinine above 150 mmol/L (OR = 2.75, 95% CI 1.69–4.50), diabetes (OR = 1.67 95% CI 1.01–2.77), liver disease, body mass index, high-risk surgery, symptomatic AAA (OR = 1.77, 95% CI 1.24–2.52), supra/juxta renal AAA (O = 2.17, 95% CI 1.32–3.57), chronic obstructive pulmonary disease (OR = 2.08, 95% CI 1.45–2.97) and smoking.

The pathophysiology of AKI is primarily the result of acute tubular necrosis caused by hypotension, hypovolemia, atheromatous embolization, rhabdomyolysis and nephrotoxins, such as contrast agents, nonsteroidal anti-inflammatory drugs and aminoglycoside antibiotics.

Specifically, the risk of AKI also varies according to the type of vascular procedure. Open elective AAA repair is associated with a mortality rate of approximately 5%, refs. [40,41,42] with the risk of developing postoperative renal failure between 1% and 6% [43,44]. The development of postoperative renal failure following elective AAA repair is a significant complication of aortic cross-clamping that causes a decrease in renal blood flow of 45% with infrarenal clamping and up to 80% with suprarenal clamping [45]. It is associated with dialysis requirement rates between 4 and 7%, a prolonged length of hospital stay and a significantly increased risk of mortality [46]. Studies specific to vascular surgical procedures also demonstrate a relationship between increased mortality and acute renal failure following emergency vascular surgery [47]. Several studies report an incidence of AKI ranging between 20% and 34% [40,41]. The incidence of AKI defined according to the RIFLE criteria and the Society for Vascular Surgery/International Society for Cardiovascular Surgery (SVS/ISCVS) reporting standards is shown in Table 1 [48].

Intraoperative strategies for renal protection are essential and include minimizing renal ischemic time, performing selective renal perfusion with cold protective solutions and using distal aortic perfusion techniques during open surgery [49,50]. Current experience suggests that cold crystalloid solutions are the best substrates in preventing ischemia-reperfusion injury. Renal perfusion using Custodiol^®^ (Dr. Franz-Kohler Chemie GmbH; Bensheim, Germany) 4 °C, even if currently considered off-label, represents an encouraging organ protection tool [51]. It is imperative to maintain an adequate intravascular volume and renal perfusion pressure throughout the operation.

EVAR has lower rates of postoperative acute renal failure despite the differences in the study designs of several trials [52,53]. In comparison to open AAA repair, EVAR engenders approximately half the risk for AKI and one-third of the risk of hemodialysis (HD) requirement [54]. Registry analyses and clinical trials, in particular the Dutch Randomized Endovascular Aneurysm Management (DREAM) trial, refined this estimate to between 0.6% and 1.1% [55,56,57,58,59]. Patients suffering postoperative AKI demonstrate a 53.5% ± 5.9% 5-year estimated survival, whereas those developing a new HD requirement exhibit 22.8% ± 8.5% 3-year estimated survival [59]. According to a recent nationwide survey, contrast media have been shown to be the leading cause of nephropathy in 6.7% of cases, especially in patients with a reduced glomerular filtration rate (Egfr < 60 mL/min/1.73 m^2^) [55]. EVAR involves the insertion and deployment of a stent-graft via the femoral artery [60], providing a less invasive alternative to open AAA repair. Randomized control trials comparing EVAR and open repair have suggested superior short-term survival with EVAR [60,61]; however, recent data have cast doubt on the long-term advantages of EVAR [62]. Although patients receiving EVAR are spared the ischemic insult of aortic cross-clamping and have less perioperative hemorrhage [63], the potential nephrotoxicity of intravenous contrast media must be considered [64]. In addition, as in the case of aortic surgery, manipulation of the abdominal aorta may lead to the disruption of lipid-laden plaques and subsequent embolization into the renal vasculature.

This observation led to consider modifiable risk factors associated with renal dysfunction, specifically the intraoperative amount of contrast medium administered. Zarkowsky et al. [65] compared the relationship between contrast medium amounts and kidney complications for elective EVAR. Of the 862 surgeons reporting cases, 447 (51.9%) performing 53.7% of all elective EVARs averaged more than 100 mL of contrast material per elective EVAR, whereas 404 (47.0%) performing 46.3% of all EVARs averaged less than 100 mL; 11 (1.3%) performing 0.08% of all EVARs reported no contrast material volume values for any cases. Interestingly, the mean intraoperative contrast medium volume was not significantly different between groups stratified by renal outcome (*p* = 0.13). On the other end, there was an association with heart failure (odds ratio [OR], 3.50; 95% CI, 1.18–10.38; *p* = 0.02) and the need to return to the operating room (OR, 3.26; 95% CI, 1.49–7.13; *p* = 0.003), whereas a preoperative eGFR above 60 mL/min/1.73 m^2^ was protective (OR, 0.33; 95% CI, 0.21–0.53; *p* < 0.001).

Normal preoperative eGFR protects against renal dysfunction. Increases in medullary filtrate viscosity stemming from contrast media contribute to nephron damage during glomerular filtration, leading to renal damage. Diminished eGFR suggests that contrast media loads will remain in contact with nephrons for a longer period at a greater concentration, further jeopardizing the function. The prevention of contrast-induced nephropathy is primarily focused on adequate hydration during and after the procedure, guidance of intravascular ultrasound and, in some cases, using carbon dioxide as an alternative contrast medium.

Preoperative renal dysfunction carries a prohibitive risk for open aortic repair surgery, and postoperative renal failure is associated with worse short- and long-term outcomes even in complex endovascular repair [66]. Early recognition of postoperative renal failure and secondary prevention of renal damage are a mainstay of intensive care unit management and biomarkers hold promise for this purpose. Recently, the combination of urinary tissue inhibitor of metalloproteinase 2 (TIMP-2) and insulin-like growth factor-binding protein 7 (IGFBP7) has performed well in the prediction of moderate to severe AKI in a mixed population of critically ill patients [67]. To date, only a few biomarkers of postoperative renal complications have been investigated in the setting of aortic repair surgery. Secretory leucocyte peptidase inhibitor (SLPI) (12 kDa) is a new possible marker to predict AKI, with a promising diagnostic accuracy at 12 and 24 h after ICU admission. SLPI is a serine protease inhibitor and is expressed by macrophages, neutrophils and many epithelial cells including the lung and kidney [68]. By inhibiting neutrophil elastase, SLPI protects proteins from digestion [69]. The half-life of serum SLPI was shown to range between 10 and 120 min [70]. Averdunk et al. [71] showed a correlation between AKI twelve hours after complex aortic intervention and a significantly increased serum SLPI, where SLPI was negatively correlated with urine output. In this trial, the ROC analysis revealed an adequate predictive accuracy of SLPI to detect AKI 12 and 24 h after admission to ICU (cut-off of 70.03 ng/mL at 12 h: sensitivity 76.47%, specificity 87.5%, AUC = 0.838; cut-off of 56.33 ng/mL at 24 h: sensitivity 75%, specificity 71.4%, AUC = 0.723). More studies are needed, and definitive clinical validation is still pending.

## 5. Neurologic Complications

Spinal cord ischemia (SCI) continues to be the most distinctive and feared complication of descending thoracic and thoracoabdominal aortic repair. 

Historically, although not accurately established, postoperative SCI has been reported in more than 20% of patients after conventional surgery and 0–13% in endovascular repair [72]. More recent surgical techniques and protective strategies have reduced the reported incidence of SCI to 3–16% [73].

The severity of the lower extremity neurological deficit can be classified, according to Greenberg, in paraplegia (no movement, minimal movement, movement not against gravity, score 0–2) or paraparesis (movement against resistance, but inability to stand, or ability to walk with assistance, score 3–4) [74]. 

The spinal cord has a complex and variable vascularization, based on one anterior and two posterior longitudinal spinal arteries that supply the anterior two-thirds and posterior third of the spinal cord, respectively. Branch vessels from the thoracoabdominal aorta form a collateral network to supply the spinal arteries. The cervical and upper thoracic portion of the spinal cord receive vessels arising from the subclavian arteries; the thoracic cord from thoracic intercostal and lumbar vessels, arising from the aorta; the lower end of the cord is supplied by branches of the lumbar, iliolumbar and sacral arteries. The segmental anterior radicular branches are frequently variable and sometimes absent and a main segmental artery, the arteria radicularis magna of Adamkiewicz, often represents the predominant blood supply to the lower two-thirds of the thoracolumbar spinal cord. This vessel arises more frequently from the left side of the aorta in the T8-L2 region. According to this vessel anatomy, interventions involving two aortic regions (zone 2, subclavian artery, and zone 5, lower thoracic artery, proximal to celiac artery) are known to have an increased risk of spinal ischemia, and the anterior portion of the spinal cord (motor function) is more frequently involved.

The pathogenesis of spinal ischemia is multifactorial, but the principal cause is the loss of spinal cord perfusion, due to surgical exclusion, embolization or hemodynamic instability. 

Many risk factors for the development of spinal injury have been investigated and classified into anatomic, perioperative and patient-specific [72,75]:

- anatomic: prior abdominal aortic surgery, number of patent lumbar arteries, extent of aortic aneurysm;
- perioperative: urgent/emergent repair, extent of coverage (total length, uncovered aorta, coverage of the left subclavian artery), endovascular landing zone 5–10 with coverage of artery of Adamkiewicz, procedure duration, general anesthesia and open surgery, hypotension, bleeding, arterial access site injury;
- patient specific: age, coronary artery disease, smoking, chronic kidney disease, chronic obstructive pulmonary disease, hypertension.

The risk of SCI has decreased since the beginning of aortic surgery, with advances in surgical technique and spinal protection protocols, even though a universally accepted protection protocol is not yet available, thus remaining a substantial institutional variability in their application [76].

Of all the interventions proposed, cerebrospinal fluid drainage (CSF) is the most studied method of spinal cord protection and the only recommended by AHA guidelines (Class 1, Evidence B) as a spinal cord protective strategy in open and endovascular thoracic aortic repair [77]. This procedure has inherent risks, and it is recommended only for high-risk repair, with a careful selection of patients, based on a clinical preoperative evaluation [77,78]. The most commonly reported criteria for a high risk of paraplegia are thoracoabdominal aneurysms type I-III, coverage of more than 15–20 cm and coverage of zone 5. The incidence of moderate to severe complications for CSF drainage placement is estimated between 7 and 9% [79,80]. 

The rationale of CSF drainage is to increase spinal cord perfusion pressure (SCPP), defined as the difference between mean arterial pressure (MAP) and CSF pressure (or central venous pressure if higher or not available). The augmentation of SCPP could prevent spinal cord injury increasing flow in case of hypotension or reduction of collateral vessels blood flow because of embolization, surgical exclusion, edema or aortic cross-clamping. 

The benefit of CSF drainage in open surgery for thoracoabdominal aortic aneurysm is confirmed in randomized controlled trials [81] and recommended by international guidelines in high-risk open and endovascular repair [77,82].

Postoperative management protocols should provide for:

- a neurological examination focused on the motor response of the lower extremities to be performed as soon as possible and to be repeated at regular intervals. If a spinal drainage is in place, a CSF pressure of 8–12 mmHg can be maintained, with a drainage of less than 20 mL per hour and no more than 40 mL during any 4 h period. If the neurological exam is normal, the drain can be closed after 24 h and removed after 48 h;
- a mean arterial pressure of 80–90 mmHg (or SCPP > 70 mmHg) in the first 48 h, using vasopressor agents if indicated. Fluid replacement must be judicious, because the increase in central venous pressure can reduce the SCPP. Be aware that many patients can have cardiac or surgical contraindications (e.g., coronary artery disease, bleeding) to the augmentation of MAP, but the concern about the SCPP is a priority during the first 48 h;
- optimal oxygen delivery, considering transfusion if the hemoglobin level is less than 10 g/dL;
- optimal coagulation with progressive rewarming of the patient;
- pharmacological treatments for the reduction of cord edema, using mannitol or glucocorticoids, may be considered but are not recommended [77].

In case of postoperative lower extremity neurological deficit:

- an immediate neurological consult should be obtained for a differential diagnosis among spinal cord ischemia, stroke or peripheral neuropathy;
- the onset of paraplegia can be immediate after surgery or delayed until the first 24–72 h (and up to several months after TEVAR). A neurological deficit on awakening from anesthesia, regardless of severity, is usually attributed to an intraoperative event causing a spinal cord infarction and is frequently associated with a poor prognosis. On the contrary, delayed onset symptoms, following a normal postoperative neurological exam, are believed to be secondary to postoperative events (hypotension, thrombosis, hematoma, embolization, high CSF pressure) and could have a better response to therapy [62];
- some protocols have been proposed for the treatment of spinal cord ischemia, all based on the further augmentation of the SCPP [1,83]. This therapy is not useful in irreversible spinal cord infarction following arterial embolization, where MAP augmentation can have deleterious effects, but it is indicated in vascular ischemia, when augmenting blood pressure may increase spinal cord perfusion. The interventions to increase SCCP are:
  ○ decrease CSF pressure to 8–10 mmHg;
  ○ increase MAP 10 mmHg every 5 min until maximum acceptable MAP is reached;
  ○ in case of no response, a lumbar drain should be placed if not already in place and CSF decreased to 8–12 mmHg;
  ○ if no response, a neurosurgery consult and spinal imaging (MRI) may be obtained to exclude epidural hematoma.

TEVAR is an attractive treatment for aneurysms, dissections, penetrating ulcers and trauma in the descending thoracic aorta, with a low periprocedural morbidity compared to open surgery [84]. TEVAR has also become a viable option for many patients with multiple comorbidities that are not eligible for open repair. 

The incidence of SCI in endovascular aortic repair is lower than in open surgical repair (2.5–8%) [85]. Some of the risk factors related to open surgery (bleeding, hemodynamic instability, general anesthesia, duration of surgery, hypothermia, damage or surgical exclusion of vessels, blood transfusion, aortic clamping, cardiopulmonary bypass, long postoperative course) do not pertain to TEVAR. In the endovascular approach, however, some risks of SCI are still present, as in open surgery, such as the anatomical variations of vessels, extent of aortic coverage, embolization, atherosclerosis, interruption of collateral vessels and prior aortic repair. Some additional peculiar risks are related to catheter manipulation, multiple or proximal sealing zones, impossibility to reimplant intercostals, contrast toxicity and post stent inflammation. In one-stage extensive TAAA repair, the incidence of SCI can be as high as in conventional open surgery [86].

## 6. Gastrointestinal Complications

Open repair portends a risk of postoperative gastrointestinal complications due to the manipulation of viscera or the ligation of visceral arteries. Ileus occurs in the first 2 or 3 postoperative days in 10 to 15% of the patients and tends to be self-limited. Direct trauma or ischemia may increase the risk of pancreatitis and cholecystitis, which are typically acalculous [15,76].

Bowel ischemia is a well-known complication following AAA repair; although it is a rare event, postoperative bowel ischemia requires a prompt diagnosis, as the associated perioperative mortality can be as high as 50% [77]. The cause of postoperative bowel ischemia is multifactorial; the role of the hypogastric arteries and their management remains disputed. The ligation or the occlusion of the hypogastric arteries during open repair is a procedure that is often performed and can be associated with ischemic complications, including spinal, pelvic and bowel ischemia. Other risk factors, including age, gender, hypertension, heart failure and surgical factors (longer operative time, extensive blood loss, suprarenal aortic cross-clamping, prolonged cross-clamp time) increase the risk of mesenteric ischemia [78].

Massive fluids resuscitation, especially after the emergent repair of a ruptured AAA, increases the risk of intra-abdominal hypertension (IAH) [79]. The deleterious effects of IAH extend beyond the damage of the intraabdominal organs (bowel, renal and liver ischemia) affecting the cardiovascular (decreased venous return, increased afterload, changes in ventricular compliance), respiratory (decreased chest wall compliance and functional residual capacity, increased peak and plateau pressures) and neurologic (increased intracranial pressure and decreased cerebral perfusion pressure). An intra-abdominal pressure above 20 mmHg associated with evidence of new organ failure defines abdominal compartment syndrome and requires emergent surgical decompression [80]. Gastrointestinal complications in endovascular procedures are less frequent compared to open surgery.

Intestinal ischemia may occur following EVAR and most commonly involves the colon, where it is reported to occur in 1–3% of patients. Although colonic ischemia after open repair is frequently profound and associated with significantly increased perioperative mortality, bowel ischemia after EVAR is frequently less severe [81].

Colonic ischemia is the result of endograft coverage of the origin of the inferior mesenteric artery: a phenomenon that occurs in all cases of EVAR of the abdominal aorta. The factors involved in this complication are the dislodgement of atherothrombotic debris resulting in microembolization to the superior mesenteric artery, inferior mesenteric artery, renal arteries, hypogastric arteries or lower extremity arterial beds [82].

Small bowel or right colonic ischemia due to hypoperfusion of the superior mesenteric artery (SMA) is much less common and may be secondary to thromboembolism from catheter and/or guidewire manipulation, especially in a long procedure or by inadvertent coverage of the SMA origin by the endograft.

Bowel ischemia is far less commonly seen in TEVAR and has been reported when there was inadvertent coverage of the celiac artery by the distal aspect of the endograft; these may often be less symptomatic if there is significant mesenteric collateralization [83]. Patients with ischemic colitis secondary to endovascular repair typically present with abdominal pain and bloody diarrhea less than 30 days after the procedure. A history of prior embolization of one or both internal iliac arteries significantly increases the risk of this complication [84,85].

## 7. Hematologic Complications

The management of coagulopathy and blood loss in aortic repair starts before surgery. Vascular patients often have multiple comorbidities that increase the risk of anemia, they are on anticoagulant or anti-platelet therapies in addition to the intraoperative administration of heparin and they undergo interventions that often involve significant blood loss. There are no specific guidelines on patient blood management for vascular surgery. We here refer to guidelines on patient blood management for adult cardiac surgery endorsed by the Task Force on Perioperative Blood Management of ASA and EACTS/EACTA [86,87]. 

Both guidelines place emphasis on the preoperative assessment, to identify patients at greater risk for bleeding and transfusion. The collection of relevant medical history, focusing on episodes of previous bleeding and innate clotting disorders, preoperative medications (e.g., clopidogrel, acetyl-salicylic acid, warfarin and other anticoagulants, vitamins or herbal supplements that may affect coagulation) and physical examination as well as laboratory tests are recommended. Activated partial thromboplastin time (aPTT), prothrombin time (PT) and a platelet count should be enough for the preoperative assessment of most patients. Extensive routine laboratory tests are unnecessary and costly and do not seem to diminish hemorrhagic perioperative complications [88]. Functional platelet tests may be reserved for patients on dual antiplatelet therapy. 

The preadmission treatment of anemia with erythropoietin and/or iron to raise preoperative hemoglobin levels should be pursued whenever possible. 

The discontinuation of anticoagulant therapy (e.g., warfarin, direct oral anticoagulants) for open, elective surgery should be planned. Antiplatelet agents such as thienopyridines should be managed according to the patient’s history of percutaneous coronary interventions [86]. It is important for all types of aortic procedures to monitor the effectiveness of intraoperative heparin-induced anticoagulation to prevent thrombosis and blood clots accumulation at sites of vascular injury. The activated clotting time (ACT) is the most used test in the intraoperative setting for unfractionated heparin (UFH) monitoring in vascular surgery [89,90]. 

Titrating heparin therapy to a target ACT between 2 and 2.5 times the baseline value (approximately 250–300 s) avoids overdose and the rebound effect in the postoperative period [91]. 

Blood should be readily available when significant blood loss is expected. The risk of bleeding differs in relation to the type of surgery: open or endovascular. 

### 7.1. Thoracic and Thoracoabdominal Open Repair

The open repair of descending thoracic and thoracoabdominal aneurysms is a high-risk procedure with a significant incidence of complications and elevated mortality. Postoperative hemorrhage occurs in 2 to 5% of patients after thoracic aortic surgery and may require surgical re-exploration, further increasing morbidity and mortality [92].

The acute coagulopathy after major vascular procedures has a multifactorial origin related to tissue injury, dilution, hypothermia, acidemia, hyperfibrinolysis and systemic inflammatory response as precipitating factors and is aggravated by shock or tissue hypoperfusion [93]. Tissue injury activates both cellular and humoral elements of the immune and coagulation systems. Proteases triggering complement release, platelet degranulation releasing phospholipid mediators, widespread endothelial disruption, increased thrombomodulin activity and Protein C activation have all been implicated [94,95,96].

The dilution of coagulation factors is now recognized as a major cause of acute coagulopathy. Reduced intravascular hydrostatic pressure during shock causes a fluid shift into the intravascular space and dilution. This is exacerbated by crystalloid use during resuscitation and red blood cells transfusion. Hypothermia inhibits coagulation protease activity and platelet function [97]. Acidosis occurs as a result of tissue hypoperfusion and excess chloride administration. It impairs plasma protease activities and coagulation factor complexes and cell surface interactions. The administration of a buffer solution does not seem to correct this coagulopathy. Other key changes in acute coagulopathy are reduction in thrombin generation, fibrinogen depletion and impaired fibrin formation. 

Extensive surgical dissection, major intraoperative blood loss and mesenteric ischemia also contribute to producing a dilutional and consumptive coagulopathy [88,98]. 

Postoperative ICU management should be focused on prevention and treatment of factors that may precipitate bleeding: normothermia; adequate volume resuscitation for hemodynamic optimization; correction of hypocalcemia. 

Evidence suggests that avoiding allogenic blood product transfusions reduces postoperative morbidity and mortality, which results in more conservative transfusion thresholds for hemoglobin (7 g/dL). In the absence of active bleeding and acute myocardial ischemia, or concern for spinal cord ischemia, a restrictive transfusion strategy to maintain hemoglobin at more than 7 g/dL is recommended [89,99].

The hemoglobin level should be increased to 10 mg/dL to maximize oxygen delivery to the ischemic spinal cord tissue and to prevent cardiac complication in high-risk patients where the trigger should be individualized. When clinical evidence for transfusion is equivocal, serial monitoring of central venous or mixed venous oxygen saturation provides an excellent assessment of tissue oxygen delivery. 

Treatment with blood components should not be delayed in order to prevent further depletion of coagulation factors and platelets. Ideally, the replacement of clotting factors, platelets and fibrinogen should be guided by standard coagulation tests (prothrombin time/international normalized ratio, partial thromboplastin time and fibrinogen) along with real-time whole blood viscoelastic tests results (thrombelastography or thromboelastometry) [100].

Additional monitoring for coagulopathy may include platelet function tests in patients with suspected or drug-induced platelet dysfunction. In the early postoperative period, thrombocytopenia may occur secondary to platelet consumption in the setting of persistent bleeding or sequestration by aortic graft material. Less commonly, the development of heparin-dependent platelet-activating antibodies may cause heparin-induced thrombocytopenia (HIT). HIT should always be considered in thrombocytopenic patients recently exposed to heparin. Later in the postoperative course, thrombocytosis and decreased fibrinolysis with hyperfibrinogenemia promote a hypercoagulable state with an increased risk of thrombotic complications requiring institution of anticoagulation (e.g., low-molecular weight heparin) [101].

Pharmacologic treatments for excessive bleeding include prophylactic antifibrinolytic therapy (tranexamic acid), desmopressin in case of platelet dysfunction, topical hemostatics (e.g., fibrin glue, thrombin gel), four-factors prothrombin complex concentrate (PCCs) in patients with excessive bleeding and increased INR, and when traditional options for treating excessive bleeding due to coagulopathy have been exhausted, consider administering recombinant activated factor VII. 

### 7.2. Abdominal Open Repair 

Intraoperative risk factors for bleeding include a long aortic cross-clamp time and large blood loss requiring massive blood transfusion and fluid replacement. Bleeding from the disruption of aortic graft anastomoses is a major surgical concern after open aortic repair. Strict blood pressure control is imperative until the risk of surgical postoperative hemorrhage is low enough to allow for a return to the patient’s preoperative hemodynamic profile. When a massive red blood cell transfusion is required, a resuscitation approach using fresh frozen plasma is beneficial in reducing the intravascular depletion of clotting factors [15].

Objective parameters from clotting assessments, such as TEG or ROTEM, are beneficial in guiding blood product administration therapy. Persistent postoperative bleeding requires prompt and aggressive treatment of hypothermia, acidosis and hypocalcemia. 

### 7.3. Endovascular Repair

Endovascular repair avoids extensive aortic dissection and aortic cross-clamping and is generally associated with reduced blood loss [102]. Endovascular stent grafting is not without risks, and complications can be immediate or delayed (beyond 1 year).

Early complications of TEVAR include vascular access injuries that occur in 15% to 20% of the cases. Frequent injuries include iliofemoral lacerations and rupture, pseudoaneurysm formation and retroperitoneal hematoma [103]. 

Blood loss in endovascular surgery may be occult and may occur at the vascular access site or anywhere along the aorta or iliofemoral arteries. The occurrence of abrupt, unexplained hypotension should prompt immediate inspection of the vascular access sites followed by a thorough angiographic survey to search for potential sources of bleeding. 

Postoperative EVAR complications are like those observed after TEVAR. Meta-analysis and review results on endovascular versus open repair of acute abdominal aortic aneurysms found that patients undergoing EVAR had a significant reduction in intraoperative blood loss compared with those undergoing open repair. Blood transfusions were significantly less likely to be required in the EVAR group, even in complex cases [104]. 

Englberger et al. showed a higher level of fibrin monomer and thrombin-antithrombin complex in patients undergoing endovascular aneurysm repair compared with the open technique, reflecting a stronger procoagulant state, probably related to endovascular manipulation [105]. 

After an uncomplicated EVAR, patients are generally admitted overnight for the purposes of observation, continuation of intravenous fluid resuscitation and monitoring of groin access sites. Complex EVARs or those performed in severely debilitated patients may necessitate a higher level of care, initiation of blood products or early postoperative cross-sectional imaging.

Access-site complications may range from a local groin hematoma to more serious arterial injuries, such as dissection, thrombosis, pseudoaneurysm, arteriovenous fistula formation, or vessel perforation or avulsion. The incidence of severe complications is quite low, less than 3%, and can be minimized by careful preoperative planning, proper device selection and judicious use of surgical conduits. 

Monitoring for bleeding complications and retroperitoneal hematoma, a conservative blood transfusion strategy unless there is evidence of organ ischemia and thromboembolic prophylaxis are the cornerstones of the management in the vascular patient subjected to EVAR [30].

## 8. Discussion

Contemporary aortic repair surgery is the result of the evolution in endovascular techniques in the past decades. Mortality for aortic aneurysm repair has declined and patients with complex aortic pathology or multiple comorbidities are now eligible for repair surgery. Major hemodynamic swings and significant blood loss are mostly avoided with an endovascular approach, but these patients are still at risk of cardiovascular complications. Postoperative cardiovascular care focuses on early recognition of myocardial ischemia, blood pressure control and euvolemic balance. Postoperative pulmonary morbidity is a major issue in open repair, and it may prohibit surgery. These patients need preoperative functional status optimization and multidisciplinary postoperative care aimed at early mobilization. Prevention of kidney injury starts with intraoperative renal protection through perfusion and contrast medium containment, while postoperative ICU care relies on early detection of failure and prevention of secondary damage. Spinal cord ischemia is the most dreaded complication of repair of the thoracic and thoracoabdominal aorta. Spinal cord protection mainly relies on CSF drainage to increase perfusion pressure during the perioperative period. A postoperative neurologic examination should be carried out at regular intervals in order to allow prompt intervention. Gastrointestinal complications other than postoperative ileus are rare but, as in the case of bowel ischemia, insidious and extremely serious. Regular examination of the abdomen is recommended and, in case of abdominal distension, monitoring of intra-abdominal pressure is required. Bleeding and coagulopathy are major issues in complex open repair either intra- or postoperatively, and point-of-care coagulation monitoring should be available to guide treatment.

Postoperative ICU care in aortic repair deserves a multidisciplinary effort of surgeons, intensive care physicians and nurses to optimize all aspects of the patient’s care. In this regard, clinical case discussions and educational activities should be regularly scheduled to reinforce clinical care processes and teamwork engagement.

In summary, aortic repair surgery has an impact on every organ system and the importance of early recognition of organ failure cannot be overemphasized.

## Figures and Tables

**Table 1:** Incidence of renal failure in aortic repair.

	*All Patients*	EVAR	*OR*
**RIFLE criteria**			
**Any AKI (No.; 95% CI)**	74% (267/362; 69–78)	63% (43/68; 51–74)	76% (224/294; 71–81)
**AKI category (No; CI)**			
**Risk**	27% (71/267; 22–32)	33% (14/43; 20–47)	25% (57/224; 20–32)
**Injury**	39% (104/267; 33–45)	40% (17/43; 26–54)	39% (87/224; 33–45)
**Failure**	34% (92/267; 29–40)	28% (12/43; 17–43)	36% (80/224; 30–42)
**If AKI, reason (No.; CI)**			
**Urine output**	44% (117/267; 38–50)	51% (22/43; 37–65)	42% (95/224; 36–49)
**SCr**	30% (80/267; 25–36)	28% (12/43; 17–43)	30% (68/224; 25–37)
**Urine output and SCr**	26% (70/267; 21–32)	21% (9/43; 11–35)	27% (61/224; 22–33)
**Days after intervention of AKI category, median (IQR)**	3 (2–4)	3 (2–4)	3 (2–4)
**SVS/ISCVS reporting standards**			
**Any AKI (No.; CI)**	48% (175/362; 43–53)	40% (27/68; 29–52)	50% (148/294; 45–56)
**AKI category (No.; CI)**			
**I**	53% (92/175; 45–60)	74% (20/27; 55–87)	49% (72/148; 41–57)
**II**	19% (34/175; 14–26)	11% (3/27; 4–28)	21% (31/148; 15–28)
**III**	28% (49/175; 22–35)	15% (4/27; 6–32%)	30% (45/148; 24–38)
**Days after intervention of AKI category, median (IQR)**	3 (2–4)	3 (2–4)	3 (2–4)

***CI**, confidence interval; **EVAR**, endovascular aneurysm repair; **IQR**, interquartile range; **OR**, open repair; **SCr**, serum creatinine*.

## Data Availability

Not applicable.
